# GCIP, Id like HLH protein, negatively regulates cell proliferation of rheumatoid synovial cells via interaction with CBP

**DOI:** 10.1186/ar3618

**Published:** 2012-02-09

**Authors:** Hidetoshi Fujita, Minako Nakazawa, Satoko Aratani, Kusuki Nishioka, Akiyoshi Fukamizu, Toshihiro Nakajima

**Affiliations:** 1 Institute of Medical Science, St Marianna University School of Medicine, Kanagawa 216-8512, Japan; 2 Advanced Radiation Biology Research Program, and Hospital, Research Center for Charged Particle Therapy, National Institute of Radiological Sciences, 4-9-1 Anagawa, Inage-ku, Chiba, 263-8555, Japan; 3 Institute of Medical Science, Tokyo Medical University, 6-1-1 Shinjyuku, Shinjyuku-ku, Tokyo, 160-8402, Japan; 4 Graduate School of Life and Environmental Sciences, University of Tsukuba, Ibaraki 305-8572, Japan

## Background

Rheumatoid arthritis (RA) is one of the most common articular diseases with a prevalence of 1% worldwide [1,2]. The clinical features of RA include chronic inflammation of systemic joints associated with synovial hyperplasia followed by impairment of quality of life [3,4]. Recently, we have shown that Synoviolin/Hrd1, an E3 ubiquitin ligase, is a novel causative factor for arthropathy [5]. However, the mechanism that regulates synovial cell outgrowth is not fully understood.

## Materials and methods

Human embryonic kidney (HEK)-293 cells, HEK-293T cells, NIH3T3 cells and synovial cells were cultured in DMEM medium. Transient transfection assays were performed in HEK-293 cells and HEK-293T cells. HEK-293 cells transfected with NF-κB-Luc were treated with 100 ng/ml of phorbol ester 12-O-tetradecanoylphorbol-13-acetate (TPA), or 10 ng/ml of TNF-α for 24 h, and luciferase activities were measured. siRNAs with 21 nucleotides for human GCIP were chemically synthesized. Transfection with siRNAs and cell survival assay were carried out.

## Results

Grap2 cyclin D interacting protein (GCIP), Id like HLH protein, was down-regulated in the rheumatoid synovial cells. Introduction of GCIP into mouse fibroblast NIH3T3 cells resulted in growth suppression, whereas knockdown with siRNAs in synovial cells enhanced cell growth. GCIP associated with CBP and repressed transcription of CREB-target genes such as cyclin D1 by inhibition of interaction between CBP and RNA polymerase II complexes. Binding assays revealed that GCIP bound to CBP via acidic region, not HLH domain, and this interaction was regulated by phosphorylation of GCIP in a cell cycle-dependent manner. Therefore, GCIP has inhibitory effect on cell proliferation via interference with CBP-mediated transcription.

## Conclusions

We propose the novel inhibitory mechanisms of Id protein family; the coactivator CBP is a functional target. Furthermore, down-regulation of GCIP may be a key factor in rheumatoid synovial cell outgrowth.
